# Leveraging drought risk reduction for sustainable food, soil and climate via soil organic carbon sequestration

**DOI:** 10.1038/s41598-019-55835-y

**Published:** 2019-12-24

**Authors:** Toshichika Iizumi, Rota Wagai

**Affiliations:** Institute for Agro-Environmental Sciences, National Agriculture and Food Research Organization, 3-1-3 Kannondai, Tsukuba, 305-8604 Japan

**Keywords:** Carbon cycle, Climate-change mitigation, Natural hazards

## Abstract

Drought is a major risk in global agriculture. Building-up soil organic carbon (SOC) enhances soil fertility and efficient use of rainwater, which can increase drought tolerance in food production. SOC management demonstrates its benefit at various locations and is a promising means to achieve food security and climate mitigation at once. However, no global assessment of its potential and co-benefits gained from SOC enhancement has been presented. Here we evaluated the extent to which SOC build-up could reduce agricultural drought risk. Using statistical analysis of spatially-explicit global crop and soil datasets, we find that relatively small enhancement in topsoil (0–30 cm) organic carbon content (OC_top_) could increase drought tolerance of the food production systems operating over 70% of the global harvested area (particularly drylands). By closing the gap between current and upper limit of tolerance levels through SOC addition of 4.87 GtC at the global scale, farmer’s economic output in drought years would increase by ~16%. This level of SOC increase has co-benefit of reducing global decadal mean temperature warming by 0.011 °C. Our findings highlight that progress towards multiple development goals can be leveraged by SOC enhancement in carbon (C)-poor soils in drier regions around the world.

## Introduction

Increasing the productivity and income of small farmers in developing world is a widely acknowledged means to achieve food security^1^. More than 570 million family farmers cultivate nearly 80% of global agricultural land and provide a dominant portion of food locally consumed^2^. Most of their lands are rainfed and susceptible to persistent drought which is the most severe climatic disaster for global food production^3^. One drought event occurred in the 1983–2009 period decreased agricultural gross domestic production by 0.8% on a global average basis, with more severe damage in developing countries than in developed countries and in drier regions than in wetter regions^4^. Droughts have also contributed to recent rise in hunger^5^. Drought risk reduction is therefore crucial to ensure the progress towards the food security goal by 2030 (ref. ^6^).

Building up SOC helps to maintain soil moisture at levels that enable crops to withstand water deficit^7–10^ because (i) soil organic matter (SOM) in which SOC acts as a backbone has enormous water holding capacity and (ii) SOM enhances the formation and stability of porous soil structure which ensures the adequate balance of moisture and aeration for plants and soil biota^2^. Environmental co-benefit of SOM includes the increase in biodiversity and nutrient storage as well as the reduction of soil erosion^11^. Various agricultural practices, such as the incorporation of manure, compost, crop residues and biochar into soil, conservation tillage, cover crop, mulching and agroforestry, have been implemented around the world to improve soil water holding capacity and fertility^8–14^. Higher crop yields in normal years^15^ and smaller yield losses in drought years^8^ are well-known benefits of these soil management.

Soil management also plays an important role for climate mitigation. Carbon sequestration potential of agricultural SOC stock is well recognized^11,16–20^ since global soils contain two to three times more C than the atmosphere^20^, and a substantial portion (37%) of the global ice-free land is in use for agriculture^21^. Recently, the dual benefits of SOC enhancement for climate mitigation and food security are emphasized in the “4 per mil initiative”^22^. Given the limitations of available C and nitrogen resources to achieve the annual increase of global soil C by 4 per mil^23–25^, it is imperative to identify the agricultural areas most suitable for additional SOC build-up in terms of the economic and environmental benefits associated with SOC increase^18^. Here we show that higher SOC content is beneficial for drought risk reduction in global food production. We examined two benefits of the SOC enhancement: (i) agricultural economic output in drought year, which has implications for small farmer’s income and access to food, and (ii) climate mitigation. We assessed four major crops (maize, rice, wheat and soybean), which together accounts for two thirds of the world’s food calories.

## Results

### Geographical pattern of drought tolerance gaps

Using the spatially-explicit global crop and soil datasets, we depict geographic pattern of drought tolerance gaps (DT_gap_**)** in landscape-level food production systems. The gap in each 0.5° grid cell is derived by comparing estimated current drought tolerance level and its attainable upper limit. For the crops, dry-year yields relative to normal yield (average yield in 1992–2008) are computed (the left panels of Supplementary Fig. S1). Their area-weighted average is used to represent the current drought tolerance level (the left lowest panel of Supplementary Fig. S1). The upper limit of drought tolerance is determined by identifying the areas of high dry-year yields within similar climate regime (the right lowest panel of Supplementary Fig. S1 and Supplementary Fig. S2a).

**Figure 1:**
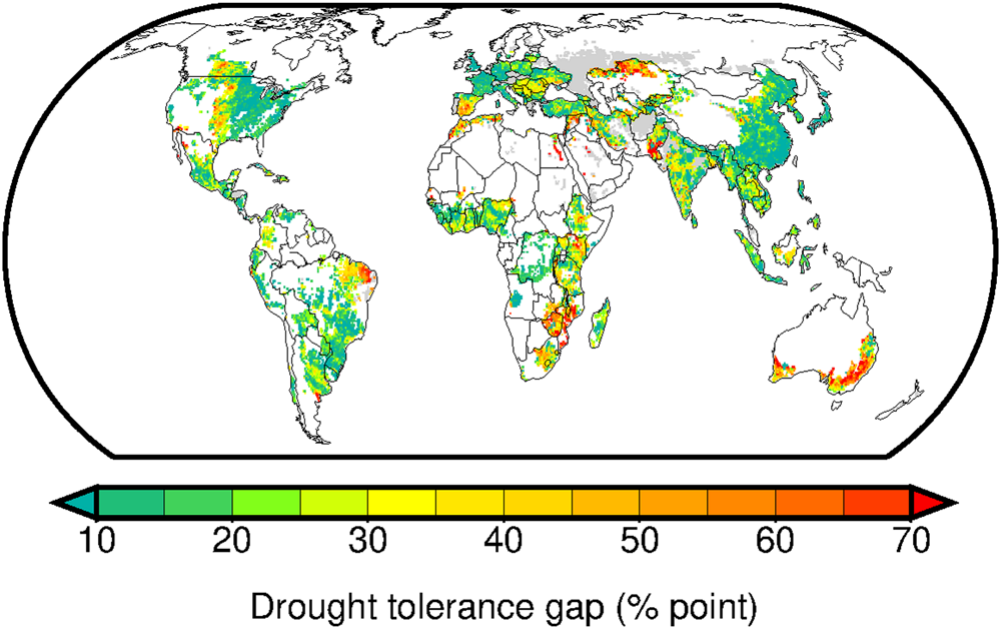
Drought tolerance gaps (DT_gap_) for maize, rice, wheat and soybean production systems circa the year 2000. DT_gap_ is indicated as the difference between dry-year average yield (% to normal yield) and its maxima within the areas under similar climate regime. Gap in each grid cell is calculated as an area-weighted average across the crops and is displayed when the harvested area of the crops is greater than 1% of the grid-cell extent. Gray color indicates the areas with no yield data.

Our analysis shows clear regional variation in the estimated DT_gap_. The areas with relatively large gap (food production is susceptible to drought) appear over drylands, including the Mediterranean, south Africa, Australia, western American Midwest and central Asia (Fig. 1). In the areas with relatively high drought tolerance (DT_gap_ is already small), the dry-year yield is only lower than the normal yield by ~10%. Such areas are distributed to eastern Asia, western Europe and eastern North America.

### Reduction of drought tolerance gaps in response to SOC increase

**Figure 2:**
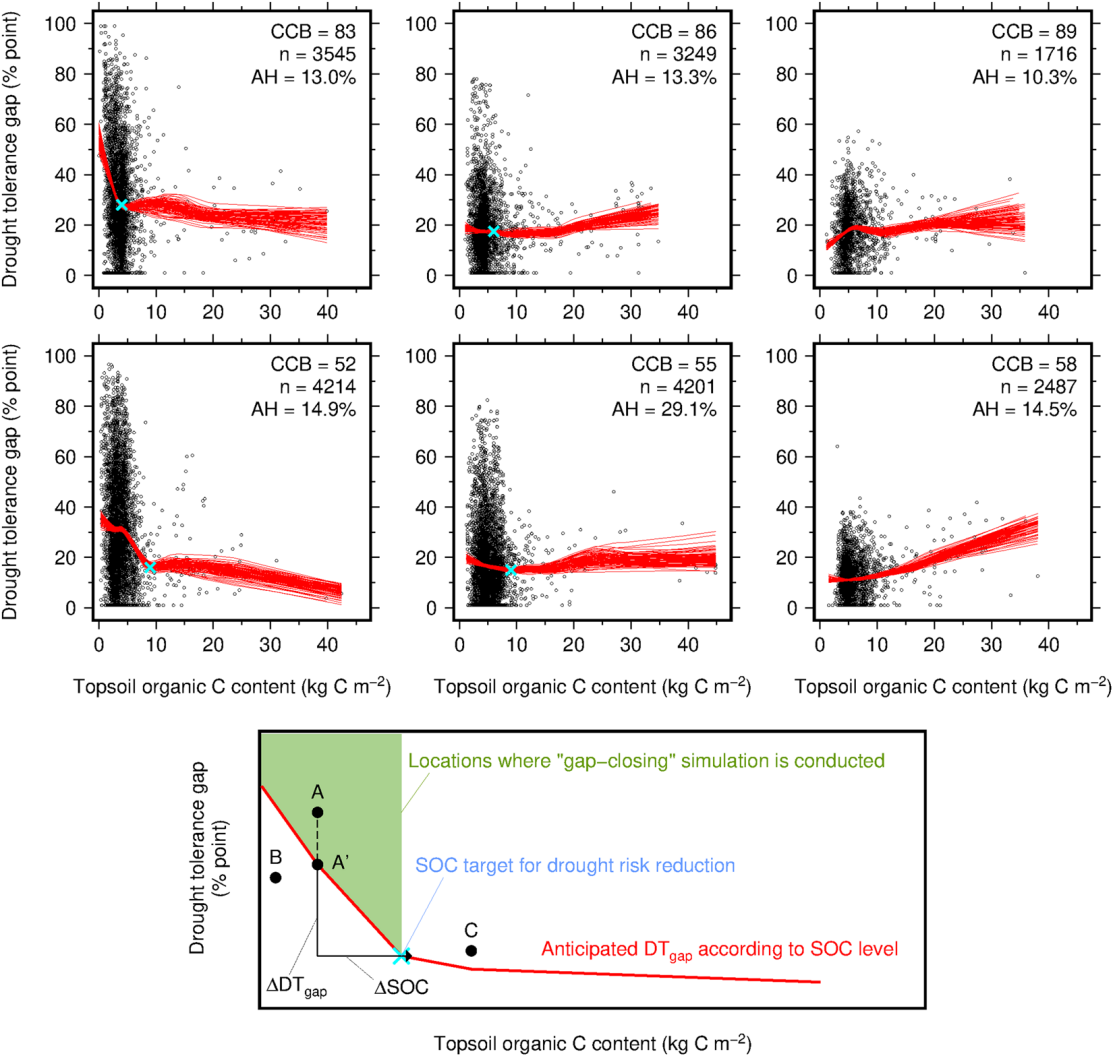
The relationships between topsoil organic carbon content versus drought tolerance gap (DT_gap_) for different climate zones (upper small panels) and schematic illustrating the “gap-closing” simulation conducted in this study (lower large panel). Each panel indicates specific climate zone; upper and lower panel indicates warmer and cooler zone, respectively; left, middle and right panel indicates drier, mildly wet and wetter zone, respectively; and the climate zones are coded by centered climate bin (CCB; Supplementary Fig. S2a). The extent of harvested areas that locate within the climate zones accounts for 95% of the global harvested area of the crops. Red lines indicate the locally weighted scatterplot smoothing (LOWESS) curves derived using 100 bootstrap replications to represent average relationships and their uncertainty; blue crosses indicate the SOC targets and corresponding DT_gap_ used in the “gap-closing” simulation; AH indicates the harvested area share by climate zone; and n indicates the sample size.

The influence of SOC level on DT_gap_ is nonlinear and C-poor soils in drier parts of the world show higher sensitivity (Fig. 2). Small increase in SOC from nearly zero to 4–9 kgC m^−2^ substantially reduces DT_gap_ in drier climate zones (the ratio between mean annual precipitation and potential evapotranspiration, P/PET <0.45). On the other hand, such effect diminishes under wetter climate. In the drier zones, average DT_gap_ sharply drops down to 16–28% point with SOC increase (red lines in CCB-52 and CCB-83 of Fig. 2). The steep decline in DT_gap_ in the drier areas is observed until the SOC content approaches 4–9 kgC m^−2^. However, further SOC increase only slightly reduces DT_gap_. The mildly wet climate zones (CCB-55 and CCB-86 of Fig. 2; 0.45 ≤ P/PET < 1.0) show the same but less-prominent trend compared to the drier zones. Furthermore, in C-rich soils (>10 kgC m^−2^), the SOC effect becomes uncertain for the mildly wet zones because data in C-rich areas is quite scarce. No reduction in DT_gap_ is found in wetter climate zones (CCB-89 and CCB-58 of Fig. 2; 1.0 ≤ P/PET). In short, the SOC effect on drought risk reduction is detected over 70.3% of the global harvested area (29.1%, 14.9%, 13.3% and 13.0% for CCB-55, 52, 86 and 83, respectively) with greater importance of moisture regime than temperature one.

Irrigation effect is hardly distinguishable at the global scale (Supplementary Fig. S3a), although the role of irrigation in moderating drought impacts is known^26,27^. Rather it appears to increase average yield level in our analysis (Supplementary Fig. S3b,c), that is consistent with ref. ^28^ which explains the causes of yield gaps. The irrigation dataset used here^29^ reports irrigation-equipped areas but not the amount of irrigation water. It is often the case that in drought years water is not available or not economically accessible for farmers. This in part explains why the relationship between irrigation intensity and DT_gap_ is scattered. Importantly, OC_top_ is only weakly correlated with irrigation intensity (Pearson’s correlation coefficient is –0.106 with p-value of <0.001 estimated using two-tailed t-test; Supplementary Fig. S3d). Consequently, the risk that the SOC effect found here covaries with irrigation effect is low.

## Discussion

Our results have implications for climate mitigation and food security as well as soil fertility. Using the relationships addressed here, we estimate that the amount of additional SOC stock required to close DT_gap_ in global food production systems is 4.87 GtC (17.53 GtCO_2_ with the 90%-probability interval of 17.29–17.84 GtCO_2_). The contributions from the mildly wet (CCB-55; 2.28 GtC) and drier zones (CCB-52; 1.94 GtC) are larger than those from the wetter zones due to their high areal coverage (29.1% and 14.9% of the global harvested area for CCB-55 and CCB-52, respectively; Fig. 2). Adequate SOC management may differ by climate zone and available labor, machinery, equipment and infrastructure. In warm regions where microbial degradation of relatively labile C (e.g., plant residue and manure) is rapid, application of chemically-recalcitrant C such as biochar is effective as long as its production and distribution system is available^30,31^. Other options, including the labile C addition, are more feasible in colder and drier environment where C degradation is slow. The corresponding CO_2_ removal from the atmosphere would avoid global decadal mean surface temperature warming of 0.011 °C, relative to preindustrial levels (1850–1900), with the uncertainty range of 0.008–0.014 °C mainly due to the variation among climate models. The estimated climate mitigation is small in absolute terms but not negligible to limit the warming to 1.5 °C as global temperature has already increase by about 1 °C (ref. ^32^).

**Figure 3:**
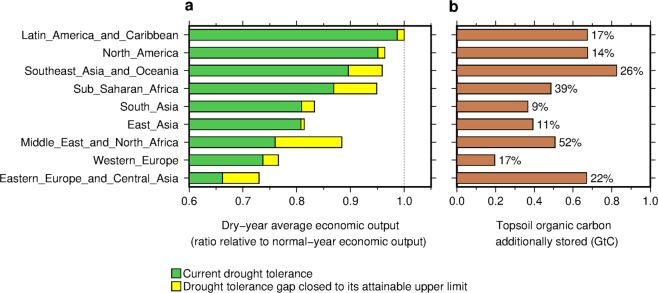
Global increases in (**a**) drought-year average annual agricultural economic output from closing drought tolerance gaps (DT_gap_) and (**b**) additional SOC required to close DT_gap_. Numbers in the right panel indicate the additional SOC in percent relative to the current regional topsoil organic carbon stock over the harvested area of the crops.

Our study highlights that, for all the regions examined here, closing DT_gap_ always increases economic outputs from food production systems in drought years (Fig. 3a) and provides a guide to target the areas where SOC enhancement is prioritized. Higher agricultural economic output would increase the income of small farmers and their access to food. In particular, relatively large increases in dry-year output are anticipated in Middle East and North Africa (a 16% increase relative to what would occur with the current DT_gap_), Eastern Europe and Central Asia (10%), Sub-Saharan Africa (9%) and Southeast Asia and Oceania (7%) (Fig. 3 and Supplementary Fig. S4). On the other hand, the regions expected to have the largest increase in SOC stock is Southeast Asia and Oceania (0.83 GtC or 26% of the current regional OC_top_ stock over the harvested area of the crops) followed by Latin America and Caribbean, North America and Eastern Europe and Central Asia (0.67 GtC each or 17%, 14% and 22%, respectively) (Fig. 3). Therefore, Middle East and North Africa and Southeast Asia and Oceania are the regions where SOC enhancement is particularly recommended for drought risk reduction. Sub-Saharan Africa and Eastern Europe and Central Asia follow these regions. Identification of the areas where SOC build-up has co-benefits for multiple development goals is critical due to the persistent challenges from nutrient source limitation, nitrogen fertilizer cost, and the competing demands for crop residues among SOC enhancement, feeding and energy^14,17,18,24^. Our study reveals that SOC build-up targeting at C-poor soils in world’s drier regions is a leverage to facilitate further progress towards multiple development goals in drought risk reduction, food security, climate mitigation and soil fertility, simultaneously.

Building up SOC may be more costly in drier regions than in wetter regions due to limited productivity and land cover, high susceptibility to soil erosion, low soil structural stability as well as high human pressure. However, with appropriate institutional arrangements, dryland holds high potential for SOC buildup through efficient water management by maximizing rainwater capture and soil water retention (e.g., rain harvesting, conservation tillage practices and selecting crops with high water use efficiency^33,34^.

A caveat is that our analysis is only relevant to the landscape scale. As indicated by the departures of many grid-cell DT_gap_ values from the average relationships (Fig. 2), factors contributing to the variation in DT_gap_ at a local scale are lacking in this study. Because yield time series data are crucial to calculate dry-year average yields and associated DT_gap_, the limited availability of high-resolution global, historical yield dataset hinders us conducting a global analysis with the spatial resolutions at which local soil characteristics play a role in characterizing DT_gap_.

## Methods

### Food production system’s drought tolerance

An indicator to represent the average tolerance of landscape-level food production systems to meteorological droughts circa the year 2000 was calculated using climate bin, growing-season precipitation and crop yields. The climate bins were defined by thermal and moisture regimes and used for climate zoning at the global scale. Maize, rice, wheat and soybean were considered. The calculation procedure consisting of three steps is described below.

### Step1: meteorological droughts

We addressed location-specific dry years during the 17 yr period centered on the year 2000 (1992–2008). Growing-season precipitation was computed for each crop, cropping season of a crop and year using the global crop calendars^35^ (the 0.5°-resolution and unfilled version) and global retrospective daily meteorological forcing dataset^36,37^ (0.5°). In the crop calendars, maize, rice and wheat have two cropping seasons (main/second for maize and rice and winter/spring for wheat), whereas soybean has one single season. For the crops with two cropping seasons, the calculated growing-season precipitation for the individual cropping seasons were averaged using the reported average production share by season in the 1990s (ref. ^38^) as the weights. Based on the calculated annual time series of growing-season precipitation of the crops, we selected the two driest years. The selected samples roughly correspond to a 10% of the sample size (n = 17) and represent years with meteorological drought more severe than the threshold for a 1-in-8.5 yr recurrence.

### Step 2: dry-year average yields

Annual yields of the individual crops in the selected dry years were averaged across the years and divided by the 17 yr average yield for the normalization (the left panels of Supplementary Fig. S1). Their area-weighted average across the crops was used as an indicator representing average food production level under meteorological drought conditions. Yield data of the crops for the period 1992–2008 were obtained from the Global Dataset of Historical Yields version 1.2 (0.5°; refs. ^39–42^; The details on the difference across versions are available in Table 1 of ref. ^42^).

Although the dry-year average yields used here represented meteorological droughts, the comparisons with the estimates of drought-induced yield losses in rainfed condition^4^ which represent agricultural droughts defined by yield reduction showed that the lower dry-year average yields accompanied the more severe drought-induced yield losses (Supplementary Fig. S6). The correlation values were low (0.134–0.293) in absolute terms but statistically significant (p < 0.001) thanks to the large sample size. This indicates that the null hypothesis that the correlation is zero was rejected; and that a positive linear relationship between the dry-year average yields and the drought-induced yield loss estimates exists. Therefore, the dry-year average yields captured to some degree the major characteristics of the estimated agricultural droughts. The drought-induced yield losses of ref. ^4^ mainly considered rainfed conditions, whereas both rainfed and irrigated conditions were considered in this study. This may in part explain their discrepancies. A comparison with other indicators of agricultural droughts would be useful, but such dataset is not available in our knowledge.

### Step 3: gap between current drought tolerance level and its upper limit

Variations in the dry-year average yield across locations within similar climatic regime must be attributed to differences in management, technology and environmental factors other than climate (e.g., soil). An approach used in ref. ^28^ offers a good analogue. For each climate bin, we selected the highest dry-year average yield value and deemed as the attainable upper limit of drought tolerance in a given climatic regime. We used climate bins shown in Supplementary Fig. S17 of ref. ^43^ which were derived based on average annual growing degree days (GDD) with the base temperature of 0 °C and average aridity index (that is, annual potential evapotranspiration of reference crop divided by annual precipitation) in 1996–2005 (Supplementary Fig. S2a). By comparing the current drought tolerance level (DT_c_ (% to average yield); the left lowest panels of Supplementary Fig. S1) and its attainable upper limit (DT_u_ (% to average yield); the right lowest panels of Supplementary Fig. S1), we derived their gap (referred to as the drought tolerance gap, DT_gap_ = DT_u_ − DT_c_ (% point); Fig. 1).

There was a concern that DT_gap_ values were largely different between the individual crops and area-weighted average due to effects of inter-crop differences in harvested area extent and yield sensitivity to precipitation deficits. However, the comparisons showed that DT_gap_ of the individual crops and their area-weighted average resembled each other (Supplementary Fig. S5). Therefore, we adopted area-weighted average DT_gap_ for the latter analysis.

### Soil organic carbon content

The topsoil (0–30 cm) organic carbon contents (OC_top_) circa the year 2000 were obtained from the regridded Harmonize World Soil Database version 1.2 (ref. ^44^). The original data at the 30-sec arc resolution was initially compiled in FAO^44^ and then aggregated the data into the 0.05° resolution^45,46^. We further aggregated the data into the 0.5° resolution. In the aggregation, we first spatially interpolated soil map at the 0.05° resolution into the 5-min arc (0.083°) resolution using the inverse distance weighted averaging method to have the common resolution with the harvested area map in 2000 (ref. ^47^). The interpolated data were aggregated to represent average level of OC_top_ over the harvested areas of the crops located within a 0.5° grid cell (Supplementary Fig. S7).

### Irrigation intensity

The extent of irrigated and rainfed areas for the individual crops were obtained from the Monthly Irrigated and Rainfed Crop Areas around the year 2000 dataset (MIRCA2000; ref. ^29^). The data at the 5-min arc resolution were aggregated into the 0.5° resolution. Then the irrigation intensity (that is, the fraction of irrigated area to harvested area) was computed crop by crop and their area-weighted average across the crops was used for the analysis (Supplementary Fig. S8).

### Average relationships between SOC and drought tolerance gap

A nonlinear relationship between OC_top_ and DT_gap_ was addressed for each of the climate zones using a locally weighted scatterplot smoothing (LOWESS) curve. The LOWESS method^48^ is powerful in depicting an empirical relationship between two variables shown in a scatter plot. We also used the bootstrap resampling technique with replacement and generated 100 bootstrap replications. The LOWESS curve was estimated for each replication using the statistical package R (lowess function with f = 0.9; R version 3.5.0; ref. ^49^) to account for the uncertainty in estimated LOWESS curves associated with data. The estimated LOWESS curves were used as the representation of average relationship and uncertainty when deriving anticipated DT_gap_ level from the current OC_top_ level. The average inflection point across the LOWESS curves (blue crosses of Fig. 2) was visually identified for each of the climate zones and used for the “gap-closing” simulation described below.

### Global SOC stock increase from closing drought tolerance gaps

To estimate the amount of additional SOC required to close DT_gap_ at the global scale, we set the SOC targets for each of the four climate zones based on the relationships addressed earlier (blue crosses in CCB-52, 55, 83 and 86 of Fig. 2). The average inflection point across the LOWESS curves was visually determined to be the SOC target (and the corresponding DT_gap_). Ths SOC targets indicate a climate-zone-specific hypothetical SOC level above which additional SOC does not help reduce DT_gap_. The remaining climate zones (CCB-58 and 89) were not included in the estimation and thus no SOC target was set because it is unlikely that SOC increases lead to closing DT_gap_ in these areas. The combinations of the SOC target and corresponding DT_gap_ used here are shown in Supplementary Table S1.

The main procedure of our “gap-closing” simulation is shown in the larger panel of Fig. 2. We selected one location (grid cell) that locates within either of the climate zones (CCB-52, 55, 83 or 86) and examined whether DT_gap_ for that location is above the anticipated level according to the current SOC level (indicated by a red line in the smaller panels of Fig. 2). If not (e.g., B in Fig. 2), the current drought tolerance level is already higher than the anticipated level and therefore no simulation was conducted for that location because no further reduction in drought risk is expected by SOC enhancement. If yes (e.g., A in Fig. 2), we compared the two data points, A’ {the current SOC level, the anticipated DT_gap_} versus the target point X {the SOC target, the corresponding DT_gap_}, and computed SOC increase and DT_gap_ reduction between A’ and X (*ΔSOC* and *ΔDT*_gap_ in Fig. 2, respectively). The average inflection point across the LOWESS curves described earlier was used as the target point. We computed *ΔSOC* and *ΔDT*_gap_ values 100 times using different bootstrap replications (Fig. 2). LOWESS curves could vary by data samples used. The 100 different curves were used to account for the uncertainty in LOWESS curve associated with data. No simulation was conducted when the current SOC level is higher than the SOC target (C in Fig. 2) because it is uncertain whether SOC increases in C-rich areas (>9 kgC m^−2^) lead to further DT_gap_ reduction (Fig. 2). These computations were repeated until all grid cells within the climate zones were completed.

### Contribution to average economic output in drought years

The calculated *ΔDT*_gap_ (and *ΔSOC*) values mentioned above were used to estimate the increases in dry-year average agricultural economic output by closing DT_gap_. For this calculation, grid-cell estimates of *ΔDT*_gap_ were first aggregated into a country scale. The grid-cell dry-year average yield when DT_gap_ is closed, *Y*_gc_ (t ha^−1^), was computed:

1 $${Y}_{{\rm{gc}},i,j}=({Y}_{i,j}-\varDelta {D}{{T}}_{{\rm{gap}},j})/100\times \overline{{Y}_{i,j}},$$

where the subscript *i* and *j* indicates crop and grid cell, respectively; *Y* indicates the dry-year average yield under the current DT_gap_ (% to normal yield); *ΔDT*_gap_ indicates the reduced DT_gap_ along with SOC increase (% to normal yield); and $\bar{Y}$ indicates the normal yield (17 yr average yield; t ha^−1^). We assumed that reduced DT_gap_ (*ΔDT*_gap_) is common across the crops. Because normal yields are different in absolute terms across the crops, a common *ΔDT*_gap_ value gives different increases in dry-year average yield in absolute terms across the crops.

The country dry-year average annual economic output from food production systems when DT_gap_ is closed, *O*_gc_ (USD), was computed:

2 $${O}_{{\rm{gc}},k}=\mathop{\sum }\limits_{i=1}^{4}\,{P}_{i,k}\times \mathop{\sum }\limits_{j=1}^{J}\,{Y}_{{\rm{gc}},i,j}\times {A}_{i,j},$$

where the subscript *k* indicates country; *A* indicates the harvested area in 2000 (ha) (ref. ^47^); *J* indicates the number of grid cells within a country; and *P* indicates the country average producer price of a crop for the period 1998–2002 (USD t^−1^). The country annual producer prices were obtained from FAO statistical database^50^. As consumer prices are more strongly affected by demand-supply balance, oil prices and others than producer prices, we used producer prices.

Additionally, the country dry-year average and normal-year economic outputs from food production systems, both under the current drought tolerance gaps, *O*_g_ and *O* (USD), were calculated:

3 $${O}_{{\rm{g}},k}=\mathop{\sum }\limits_{i=1}^{4}\,{P}_{i,k}\times \mathop{\sum }\limits_{j=1}^{J}\,{Y}_{{\rm{g}},i,j}\times {A}_{i,j},$$

and

4 $${O}_{k}=\mathop{\sum }\limits_{i=1}^{4}\,{P}_{i,k}\times \mathop{\sum }\limits_{j=1}^{J}\,\overline{{Y}_{i,j}}\times {A}_{i,j}.$$

For visualization purpose, these values were normalized using the normal-year economic output as the base (*O*_gc,*k*_/*O*_*k*_ and *O*_g,*k*_/*O*_*k*_; Supplementary Fig. S4). We also calculated increases in country dry-year average annual economic output from closing DT_gap_ relative to what would occur with the current DT_gap_ (*O*_gc,*k*_/*O*_*g*,*k*_). The calculated country estimates of dry-year economic output increase (and SOC stock increase) from closing DT_gap_ were aggregated into nine regions using the classification of regions shown in Supplementary Fig. S9. The regional aggregation of *O*_gc,*k*_/*O*_*k*_ and *O*_g,*k*_/*O*_*k*_ was indicated by yellow bars and green bars of Fig. 3a, respectively. The regional SOC stock increase computed based on *ΔSOC* is shown in Fig. 3b.

### Contribution to climate mitigation

The estimated amount of additional SOC stock over the global cropland topsoil in GtC (aggregated from *ΔSOC*) was first converted into a value in GtCO_2_ and further converted into a global decadal mean surface temperature change relative to 1850–1900. A liner relationship between cumulative total anthropogenic CO_2_ emission from 1870 and global decadal mean surface temperature change relative to 1861–1880 is found in Figure SPM.10 of Intergovernmental Panel on Climate Change (IPCC) Working Group I Fifth Assessment Report^51^. We recalculated this relationship using the bias-corrected daily mean 2-m air temperature data of eight atmosphere-ocean coupled general circulation models (GCMs)^37,52^ used in the Coupled Model Intercomparison Project phase 5 (CMIP5)^53^ (Supplementary Table S2). Cumulative CO_2_ emission was calculated using the reported emission estimates from fossil-fuel burning, cement manufacture and gas flaring for the period 1870–2009 (ref. ^54^) and Representative Concentration Pathways (RCPs) for the period 2010–2100 (ref. ^55^). As a result, we obtained the warming rate of 6.376 × 10^−4^ °C (GtCO_2_)^−1^ in global decadal mean surface temperature relative to 1850–1900 with the 90%-probability interval from 4.482 × 10^−4^ to 7.898 × 10^−4^ °C (GtCO_2_)^−1^ associated with use of different GCMs (Supplementary Fig. S10). This modification was to shift the reference time interval from 1861–1880 used in ref. ^43^ to 1850–1900 because the latter is utilized in the recent IPCC special report^32^ and enables us deriving a more robust estimate of global temperature in preindustrial levels. The same approach is found in ref. ^43^.

## Supplementary information

Supplementary Tables and Figures

Supplementary Information

## Data Availability

The data that support the findings of this study are available in Supplementary Information.
