# Supplementary material for: Leveraging drought risk reduction for sustainable food, soil and climate via soil organic carbon sequestration
Source: Sci Rep. 2019 Dec 24;9:19744. doi: 10.1038/s41598-019-55835-y (PMC6930204; doi:10.1038/s41598-019-55835-y)
Supplement: Supplementary file 1 — Supplementary Tables and Figures [file 41598_2019_55835_MOESM1_ESM.pdf]

Supplementary Information

Leveraging drought risk reduction for sustainable food, soil and climate

Toshichika Iizumi & Rota Wagai

Supplementary Table S1. The SOC targets and corresponding drought tolerance gaps (DT_{gap}) for different climate zones. The data are the same with those presented in Fig.2 (blue crosses). No “gap-closing” simulation is conducted for CCB-58 and 89.

Climate zones	SOC target (kgC m^{-2})	Corresponding DT_{gap} (% point)
CCB-52	9.0	16.0
CCB-55	9.0	15.0
CCB-58	N.A.	N.A.
CCB-83	4.0	28.0
CCB-86	6.0	17.5
CCB-89	N.A.	N.A.

Supplementary Table S2. List of the GCMs, modeling groups obtained from the CMIP5 multimodel ensemble dataset[54]. The temperature sensitivity calculated using the bias-corrected CMIP5 GCM dataset[38, 53] and utilized for this study are also shown. The temperature sensitivity indicates global decadal mean surface air temperature change relative to 1850–1900 in response to one $GtCO_2$ change in the atmosphere.

GCM name	Modeling group	Temperature sensitivity (10^{-4} °C $(GtCO_2)^{-1}$)
GFDL-ESM2M	NOAA Geophysical Fluid Dynamics Laboratory	4.482
IPSL-CM5A-LR	Institut Pierre-Simon Laplace	7.390
HadGEM2-ES	Met Office Hadley Centre	7.825
MIROC-ESM-CHEM	Japan Agency for Marine-Earth Science and Technology (JAMSTEC), Atmosphere and Ocean Research Institute (AORI) (The University of Tokyo), and National Institute for Environmental Studies (NIES)	7.898
MIROC-ESM		7.631
MIROC5	AORI (The University of Tokyo), NIES, and JAMSTEC	5.478
MRI-CGCM3	Meteorological Research Institute	5.014
NorESM1-M	Norwegian Climate Centre	5.288

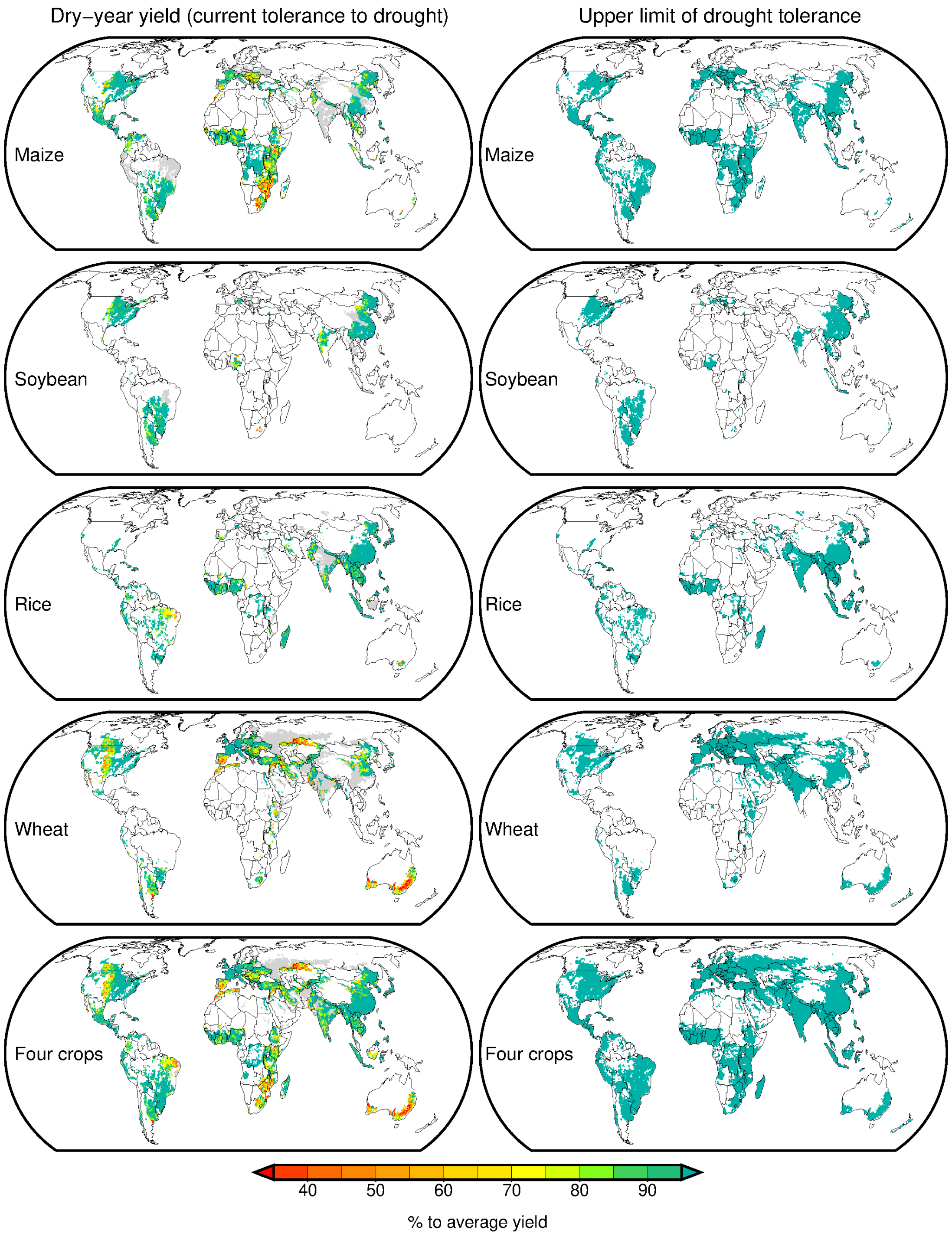

Supplementary Figure S1. The dry-year average yields of the individual crops circa the year 2000 (left; DT_c) and the attainable upper limit of drought tolerance (right; DT_u) determined by identifying areas with high dry-year average yield within areas of similar climate. The dry-year average yields correspond to the current drought tolerance level. Dry-year average yield in each grid cell is displayed when harvested area of a crop of interest is greater than 1000 ha. Area-weighted average across the crops is displayed when harvested area of the crops is greater than 1% of the grid-cell extent (~3088 ha). Gray area indicates that yield data are lacking.

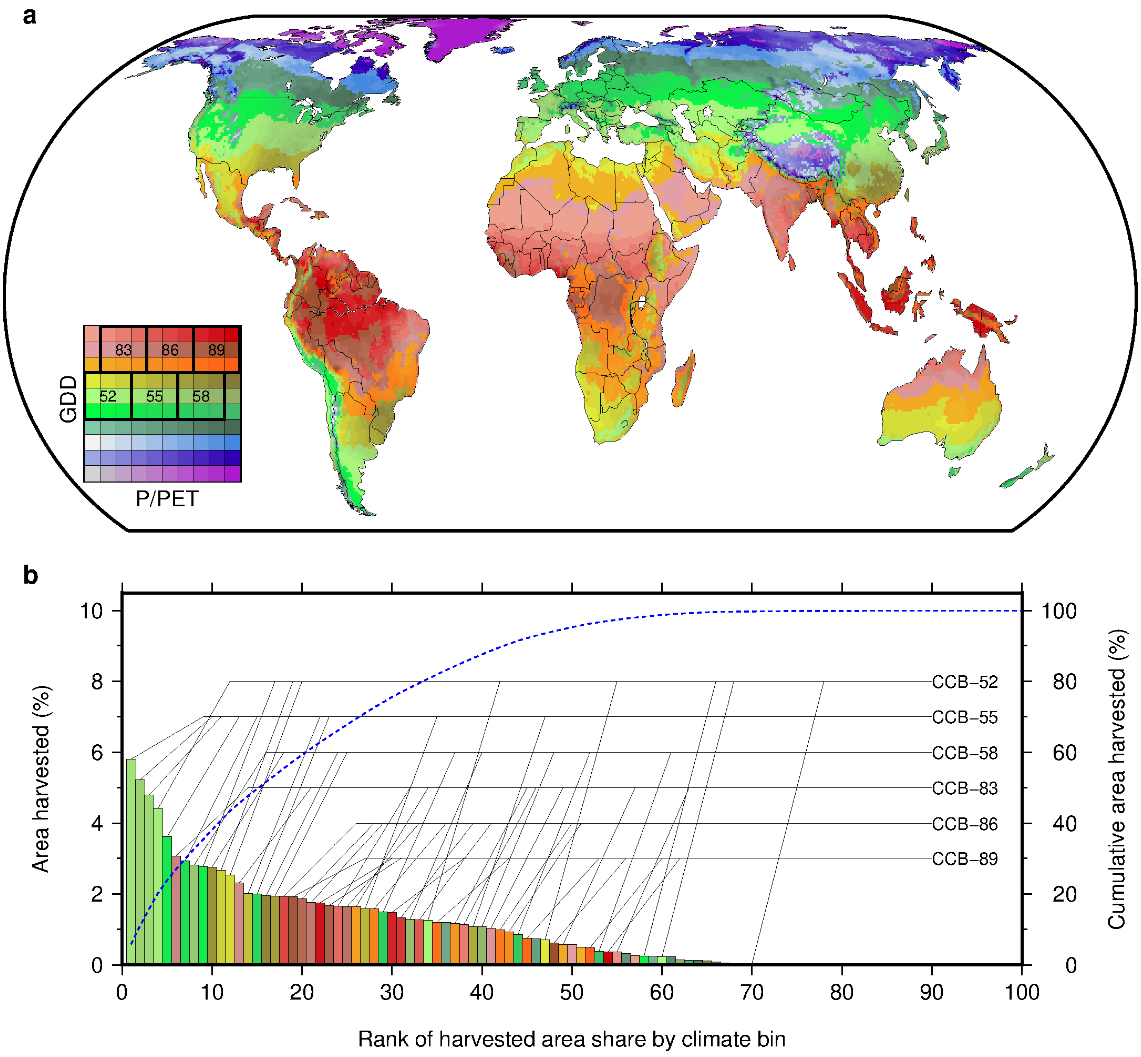

Supplementary Figure S2. The geographic pattern of climate bins and six climate zones examined in this study which account for 95% of the global harvested area of the crops (a) and the harvested area share by climate bin (and by climate zone) and their accumulation (b). Each climate zone consists of nine different climate bins and coded by centered climate bin (CCB). The climate bin map data are taken from ref. 44.

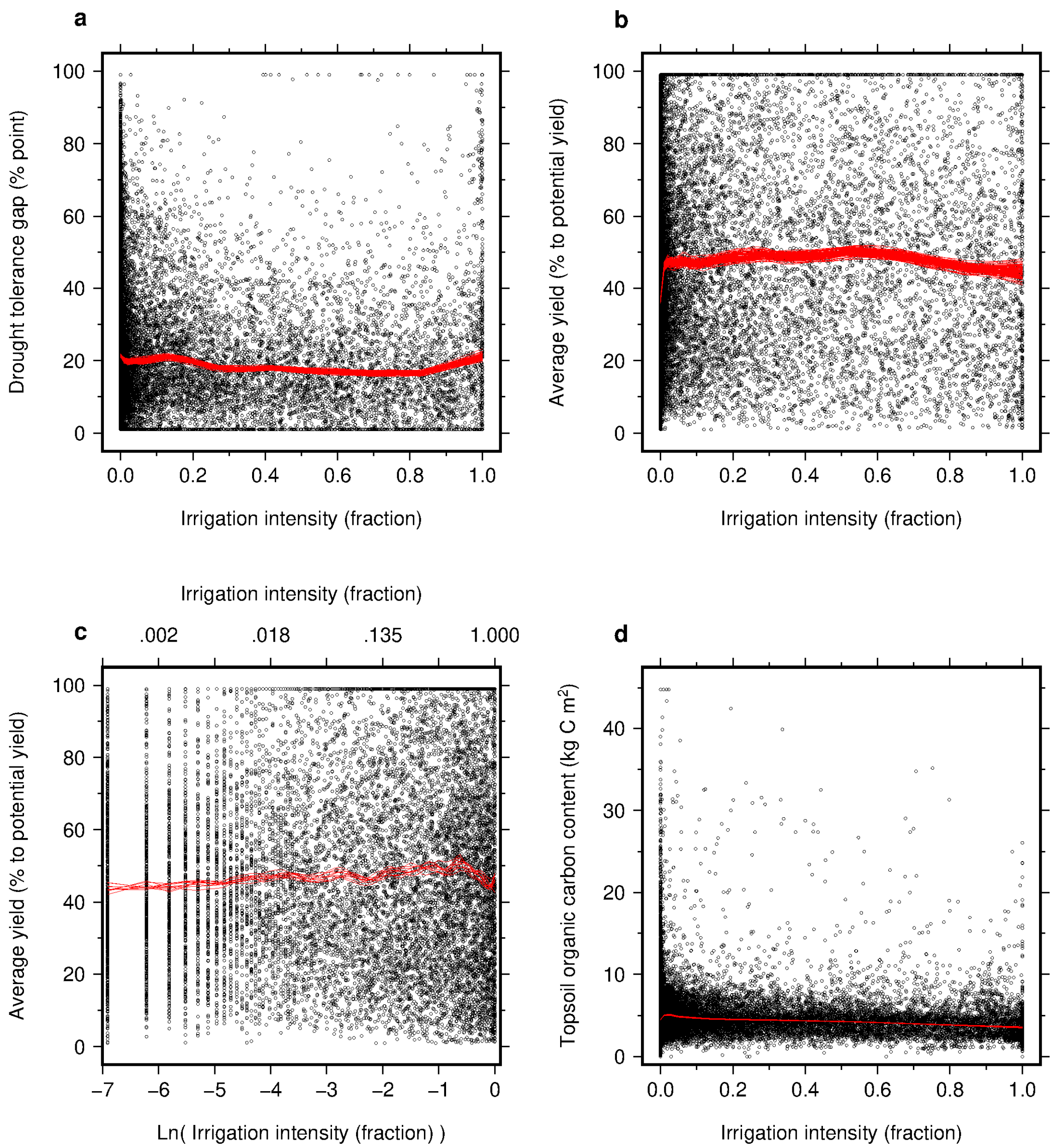

Supplementary Figure S3. The relationship between (a) irrigation intensity versus drought tolerance gap; (b) irrigation intensity versus average yield (normalized relative to attainable yield obtained from ref. 41); (c) natural logarithm of irrigation intensity versus average yield; and (d) irrigation intensity versus topsoil organic carbon content. Red lines indicate the LOWESS curves derived using 10 bootstrap replications to represent average relationships and their uncertainties.

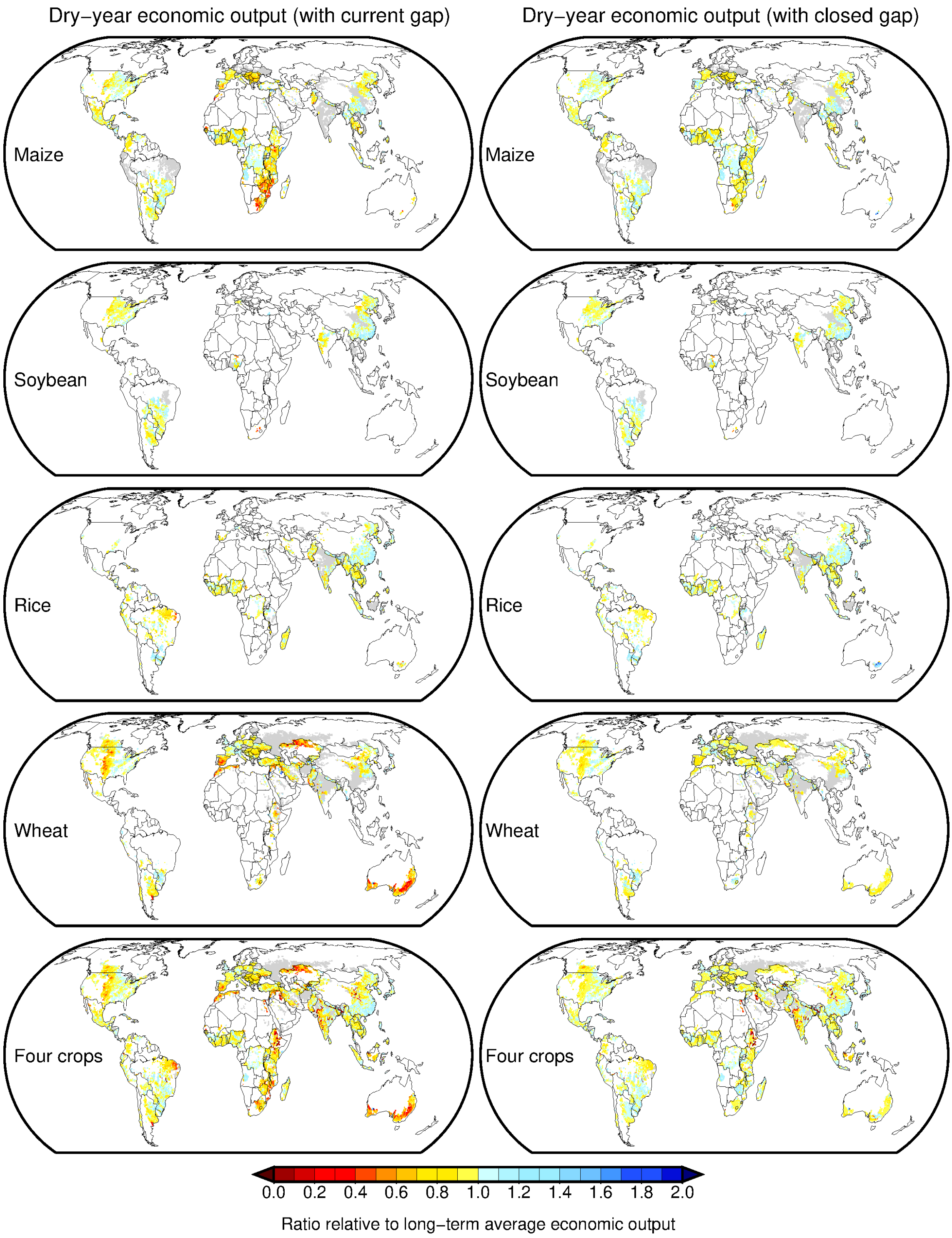

Supplementary Figure S4. Geographic patterns of (left) dry-year average annual economic output for the individual crops and their aggregation under the current drought tolerance gaps (DT_{gap}) and (right) those when DT_{gap} are closed. Data are expressed relative to normal-year economic output. Gray area indicates that yield data are lacking.

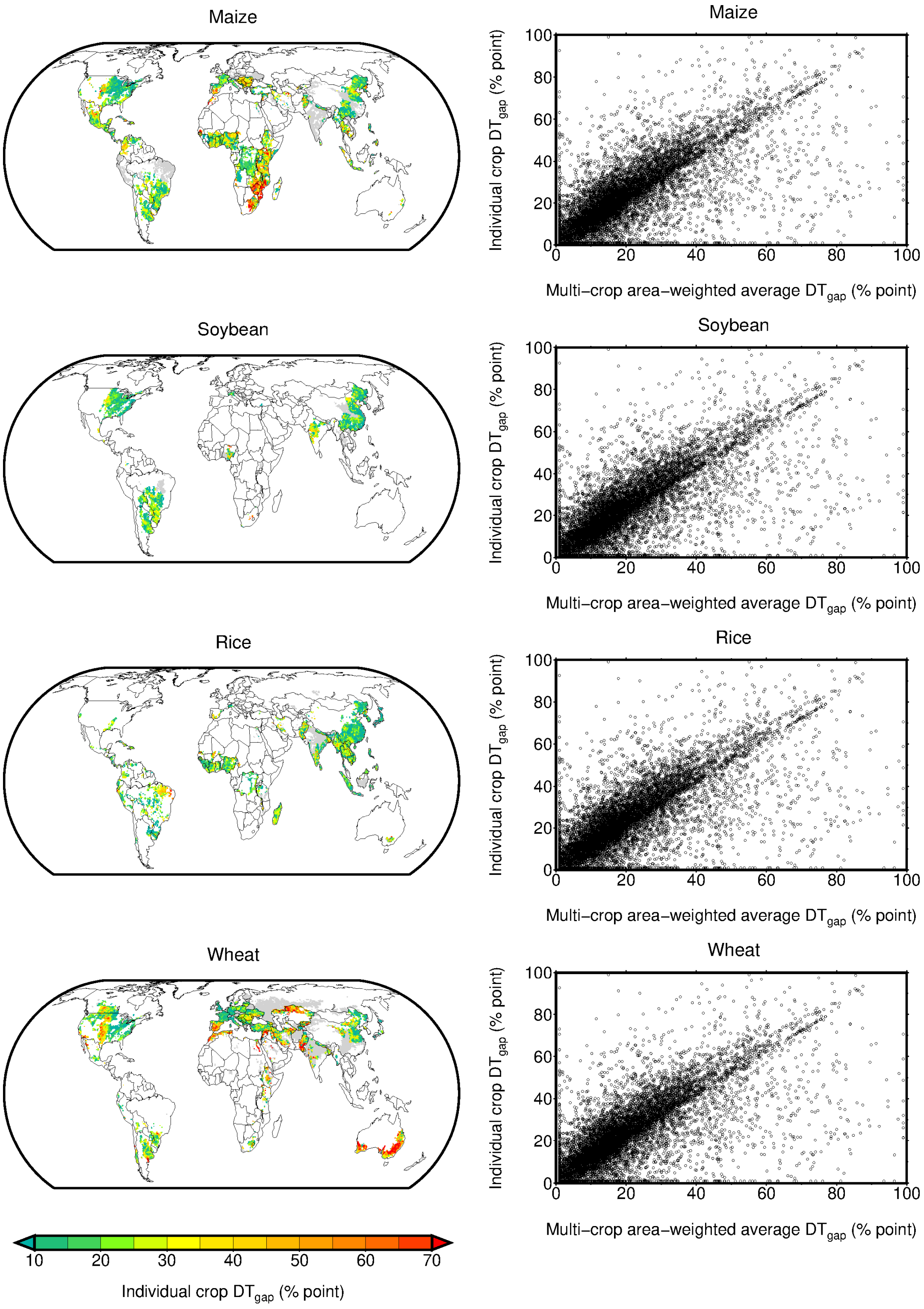

Supplementary Figure S5. The geographic pattern of the drought tolerance gaps (DT_{gap}) of the individual crops (left) and the comparisons between DT_{gap} of the individual crops and the multi-crop area-weighted average DT_{gap} (right). Gray area indicates that yield data are lacking.

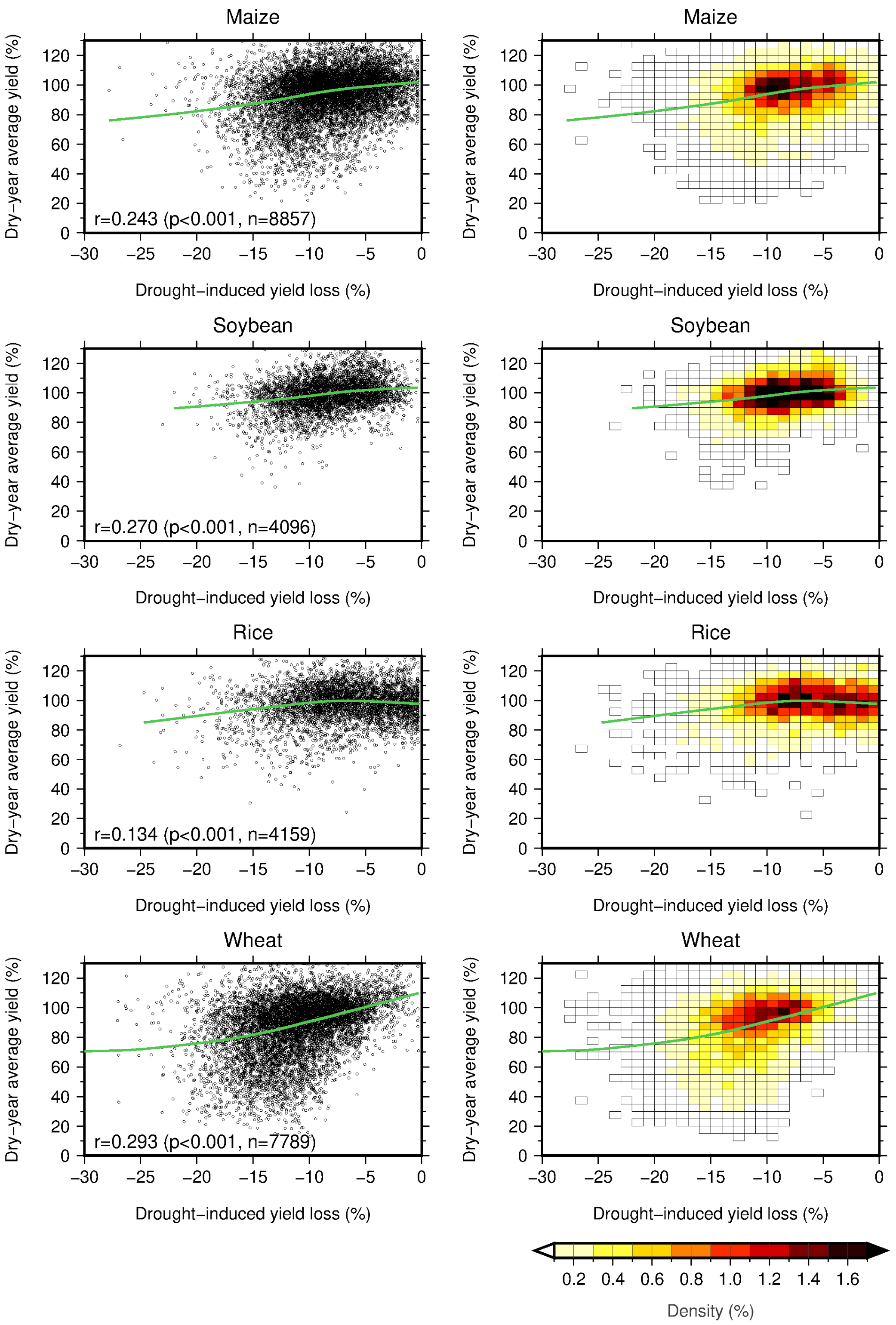

Supplementary Figure S6. Comparisons between the dry-year average yields and drought-induced yield losses in rainfed condition of ref. 4 for the four crops (left) and their smoothed density scatter plots (right). Green lines indicate the LOWESS curves to represent average relationships between the two variables in the scatterplots.

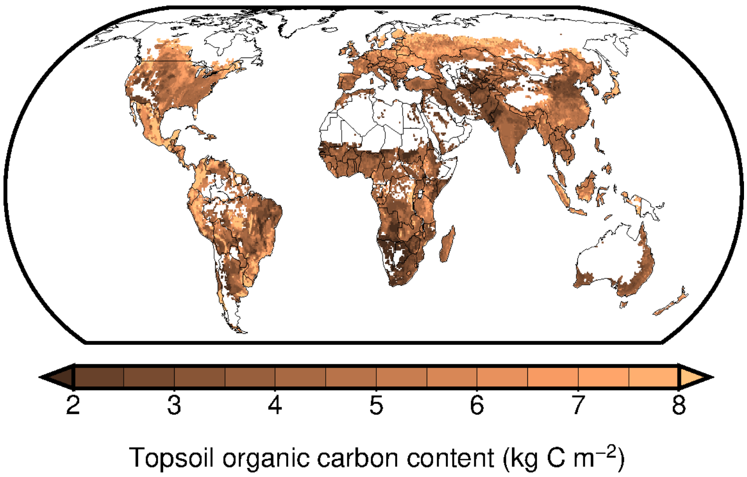

Supplementary Figure S7. The topsoil organic carbon content over the global harvested area of the crops circa the year 2000. The data are based on ref. 47.

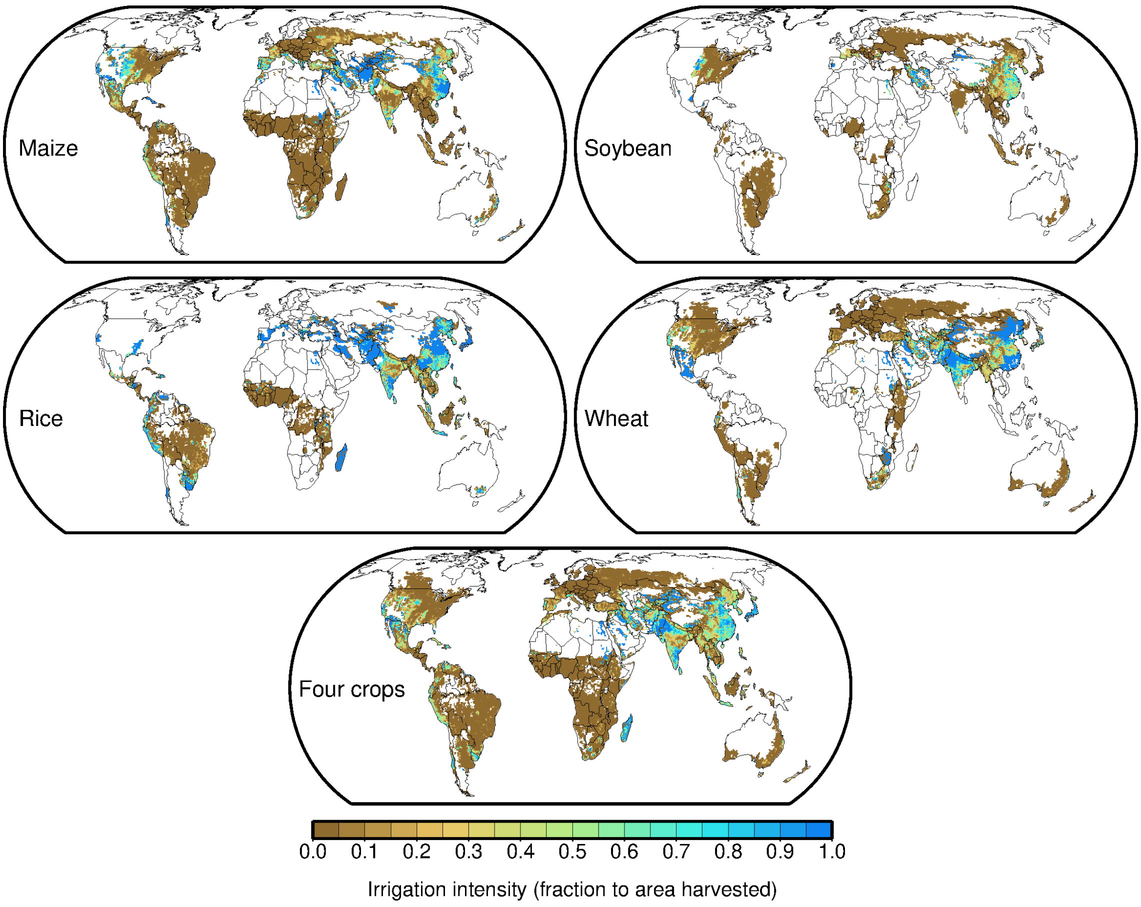

Supplementary Figure S8. The irrigation intensity of the individual crops and their area-weighted average circa the year 2000. The data are based on ref. 29.

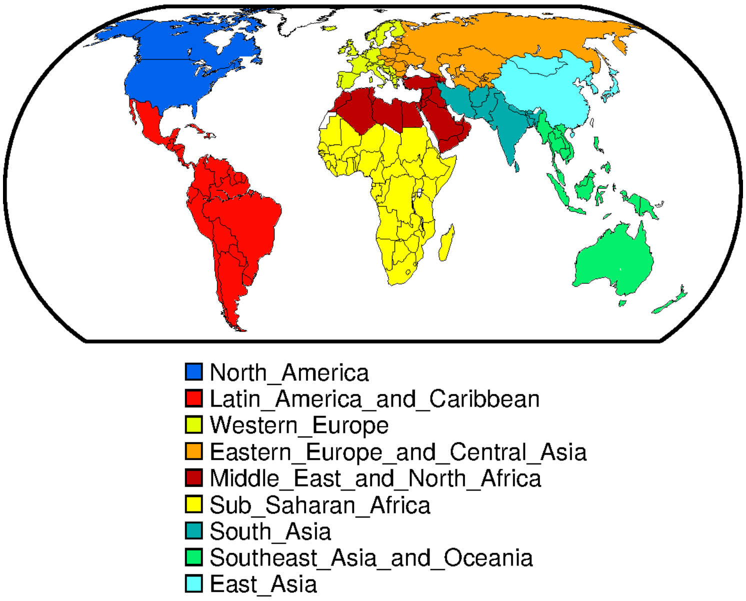

Supplementary Figure S9. The classification of world regions used in this study.

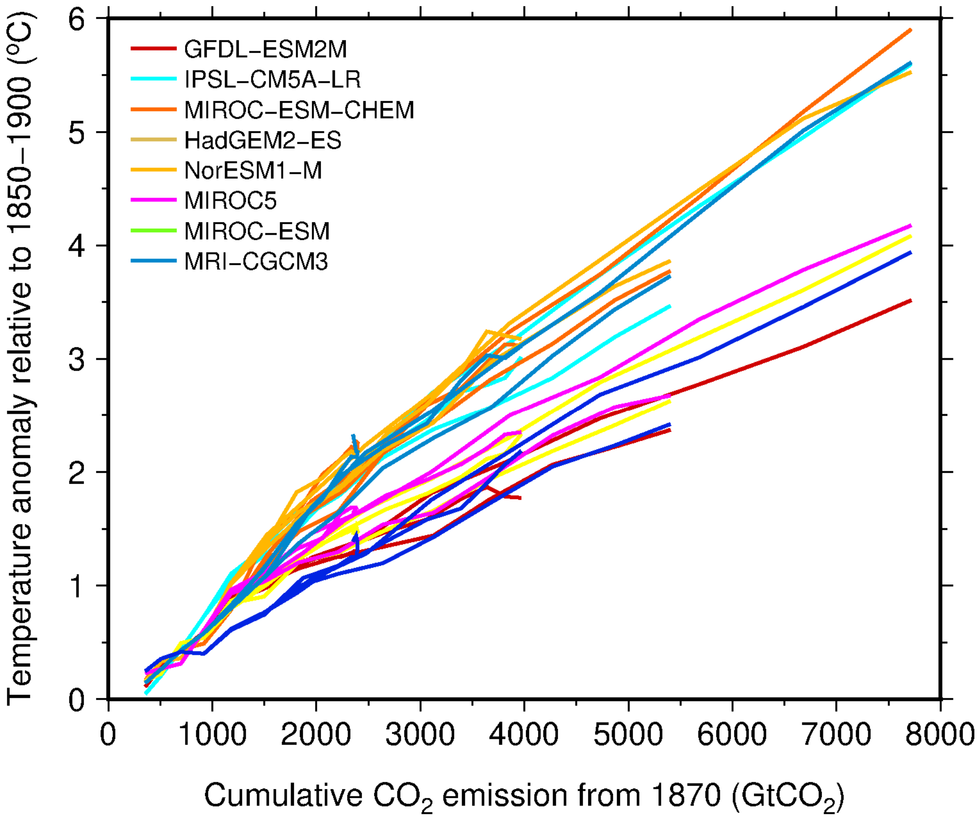

Supplementary Figure S10. Global decadal mean surface temperature anomaly relative to preindustrial period (1850–1900) as a function of cumulative total global CO_2 emissions from 1870. A colored line indicates one climate projection for the period 1961–2100 derived from a combination of GCM and RCP (the HadGEM2-ES data were only available for the 1961–2098 period). Thirty-two combinations consisting of eight GCMs (Supplementary Table S2) and four RCPs (2.6, 4.5, 6.0 and 8.5 W m^{-2}) are shown.
